# In vitro study of uptake and synthesis of creatine and its precursors by cerebellar granule cells and astrocytes suggests some hypotheses on the physiopathology of the inherited disorders of creatine metabolism

**DOI:** 10.1186/1471-2202-13-41

**Published:** 2012-04-26

**Authors:** Claudia Carducci, Carla Carducci, Silvia Santagata, Enrico Adriano, Cristiana Artiola, Stefano Thellung, Elena Gatta, Mauro Robello, Tullio Florio, Italo Antonozzi, Vincenzo Leuzzi, Maurizio Balestrino

**Affiliations:** 1 Department of Experimental Medicine, La Sapienza Università di Roma, Viale del Policlinico 155, Rome 00161, Italy; 2 Department of Neuroscience, Ophthalmology and Genetics, University of Genova, Via De Toni, 5, Genoa 16132, Italy; 3 Department of Oncology, Biology and Genetics, University of Genova, Largo Rosanna Benzi, 10, Genoa 16132, Italy; 4 Department of Physics, University of Genova, Via Dodecaneso, 33, Genoa 16146, Italy; 5 Dept of Child Neurology and Psychiatry, La Sapienza Università di Roma, Via dei Sabelli 108, Rome 00185, Italy; 6 Department of Molecular Medicine, La Sapienza Università di Roma, Viale Regina Elena 324, Rome 00161, Italy

**Keywords:** L-arginine:glycine amidinotransferase (AGAT), Creatine transporter (CT1), S-adenosylmethionine: guanidinoacetate N-methyltransferase (GAMT), Energetic metabolism in CNS, Mass spectrometry, Creatine transporter gene (SLC6A8)

## Abstract

**Background:**

The discovery of the inherited disorders of creatine (Cr) synthesis and transport in the last few years disclosed the importance of blood Cr supply for the normal functioning of the brain. These putatively rare diseases share a common pathogenetic mechanism (the depletion of brain Cr) and similar phenotypes characterized by mental retardation, language disturbances, seizures and movement disorders. In the effort to improve our knowledge on the mechanisms regulating Cr pool inside the nervous tissue, Cr transport and synthesis and related gene transcripts were explored in primary cultures of rat cerebellar granule cells and astrocytes.

**Methods:**

Cr uptake and synthesis were explored in vitro by incubating monotypic primary cultures of rat type I astrocytes and cerebellar granule cells with: a) D_3_-Creatine (D_3_Cr) and D3Cr plus β-guanidinopropionate (GPA, an inhibitor of Cr transporter), and b) labelled precursors of Guanidinoacetate (GAA) and Cr (Arginine, Arg; Glycine, Gly). Intracellular D3Cr and labelled GAA and Cr were assessed by ESI-MS/MS. Creatine transporter (*CT1*), L-arginine:glycine amidinotransferase (*AGAT*), and S-adenosylmethionine:guanidinoacetate N-methyltransferase (*GAMT*) gene expression was assessed in the same cells by real time PCR.

**Results:**

D3Cr signal was extremely high in cells incubated with this isotope (labelled/unlabelled Cr ratio reached about 10 and 122, respectively in cerebellar granule cells and astrocytes) and was reduced by GPA. Labelled Arg and Gly were taken up by the cells and incorporated in GAA, whose concentration paralleled that of these precursors both in the extracellular medium and inside the cells (astrocytes). In contrast, the increase of labelled Cr was relatively much more limited since labelled Cr after precursors' supplementation did not exceed 2,7% (cerebellar granule cells) and 21% (astrocytes) of unlabelled Cr. Finally, *AGAT, GAMT *and *SLC6A8 *were expressed in both kind of cells.

**Conclusions:**

Our results confirm that both neurons and astrocytes have the capability to synthesize and uptake Cr, and suggest that at least in vitro intracellular Cr can increase to a much greater extent through uptake than through *de novo *synthesis. Our results are compatible with the clinical observations that when the Cr transporter is defective, intracellular Cr is absent despite the brain should be able to synthesize it. Further research is needed to fully understand to what extent our results reflect the in vivo situation.

## Background

The discovery of the inherited disorders of Cr metabolism has significantly improved the knowledge on the role of creatine (Cr) in the human brain energetic metabolism [1,2] through the observation of the clinical consequence of Cr deficiency. These putatively rare diseases are due either to defects of the enzymes devoted to Cr synthesis (L-arginine:glycine amidinotransferase (AGAT, EC 2.1.4.1) [3] and S-adenosylmethionine:guanidinoacetate N-methyltransferase (GAMT, EC 2.1.1.2) [4]) - or to defects of the Cr transporter (CT1) [5,6]. They all share a common pathogenetic mechanism (the depletion of brain Cr) and similar phenotypes characterized by mental retardation, language disturbances, seizures and movement disorders. The clinical symptoms in AGAT and GAMT deficiencies are partially reversed by oral Cr supplementation [7,8], and possibly prevented by an early onset treatment [9,10], which also restores almost totally the brain Cr signal [11]. On the contrary, no effective treatment is available for the defect of CT1: while the supplementation of the precursors of Cr, Arginine (Arg) and Glycine (Gly), restores the Cr synthesis in peripheral cells in vitro [12], it results in only a mild clinical improvement with scarce [13] or absent [14] increase of brain Cr signal in vivo. So these rare metabolic disorders disclosed the importance of blood Cr supply for the normal functioning of the brain, even though AGAT and GAMT are widely expressed in the nervous tissue [15] where Cr synthesis certainly occurs [16,17].

It has been hypothesized that in the absence of CT1, neurons can not take up glial-synthesized Cr or guanidinoacetate (GAA), so suggesting a glia-neuron interplay in brain Cr homeostasis [18]. According to this model, we would expect a different distribution of the components involved in Cr metabolism (transport and synthesis) in neurons and glial cells. In the effort to improve our knowledge on the mechanisms regulating Cr pool inside the nervous tissue, Cr synthesis was explored in primary cultures of rat cerebellar granule cells and astrocytes by using Arg and Gly labelled by stable isotopes. Since these compounds are substrates of AGAT (Arg and Gly) and GAMT (GAA), the detection of the labelled products makes it possible to speculate on the distribution of these enzymes in different cells. Moreover, the role of CT1 was tested by incubating cerebellar granule cells and astrocytes with labelled Cr in the presence and absence of guanidinopropionate (GPA), a specific inhibitor of CT1. Finally CT1 (SLC6A8), GAMT and AGAT gene expression was investigated by the identification and quantification of their transcripts in the same kind of nervous cells in comparison with several other tissues.

## Methods

### Chemicals and reagents

D_3_-Creatine (D3Cr) was purchased from CDN isotopes (Quebec, Canada). ^13 ^C_2_-Guanidinoacetic acid ([1,2-^13 ^C_2_]GAA) was purchased from Dr. Herman J. ten Brink (Amsterdam, Netherlands). L-[guanido-^15 ^N_2_]arginine:HCl ([^15 ^N_2_]Arg) and [^13 ^C_2_,^15^N]glycine ([^13 ^C_2_,^15 ^N]Gly) were obtained from Cambridge Isotopes Laboratories Inc. (Massachusetts, USA). GPA, Gly, Arg, GAA, and Cr were obtained from Sigma-Aldrich (St Luis, MO, USA). HPLC grade methanol and formic acid were obtained from Merck (Darmstadt, Germany). 3 N HCl in n-butanol solution was purchased from Regis Technologies Inc (Morton Grave, USA). All solutions were prepared using highly purified water produced by a Milli-RO/Milli-Q system (Millipore, Bedford, Ma, USA). Centricone filters were obtained from Millipore (Millipore, Bedford, Ma, USA).

Wistar rats were obtained from Harlan Italy, S. Pietro al Natisone Udine. Twenty mm poly-l-lysine-coated glass coverslips were obtained from Warner Instruments LLC (Hamden, CT USA). Basal Eagle's culture medium, fetal calf serum, gentamicin and cytosine arabinoside were obtained from Sigma Aldrich (St Luis, MO, USA). PBS containing trypsin, bovine serum albumin, DMEM, fetal bovin serum, penicillin/streptomycin, L-glutamine were obtained from Euroclone (Pero - Milano Italy).

### Rat cell culture

Cerebellar granule cells were prepared from 8-day-old Wistar rats as previously described [19]. The cells were plated at a density of 1 × 10^6 ^per dish on 20 mm poly-l-lysine-coated glass coverslips and maintained in Basal Eagle's culture medium, containing 10% fetal calf serum, 100 μg/ml gentamicin and 25 mmol/L KCl, at 37°C in a humidified 95% air, 5% CO_2 _atmosphere. Cultures were treated with 10 μmol/L cytosine arabinoside from day 1 in order to minimize proliferation of non-neuronal cells. Primary cultures of rat type I astrocytes were established as previously reported [20]. Briefly, two-day-old Wistar rats were sacrificed by decapitation; brain cortices were rapidly removed and separated from meninges. Brains were minced and incubated in PBS containing trypsin (0.125% at 37°C. for 15 min). Digestion was stopped adding PBS containing 1% bovine serum albumine and was followed by mechanical trituration. The obtained cell dispersion was plated into 75 cm^2 ^flask and cultured in DMEM supplemented with 10% fetal bovin serum, penicillin/streptomycin (10 mmol/L), L-glutamine (2 mmol/L) and maintained in 5% CO_2 _atmosphere at 37°C. At confluence, flasks were shaken in rotation plates to remove microglia and type II astrocytes. Adherent cells were then divided (3:1 ratio) to grow a P1 generation. From confluent, P1 flasks was obtained a P2 generation of astrocytes that was used for most experiments. In some cases, a third (P3) generation was obtained, and enzyme expression was compared in the 3 (P1, P2 and P3) astrocyte generations. All animal experiments were carried out with authorization by the Italian ministry of Health and in compliance with the animal care requirements that are requested by Italian law (law D.L. 27.1.1992 n. 116, in agreement with the European Union directive 86/609/CEE).

### Cell treatment

For Cr uptake experiments, rat cerebellar granule cells and astrocytes were incubated for 24 hours in a culture medium containing1 mmol/L D3Cr and with 1 mmol/L D3Cr plus 1 mmol/L GPA. D3Cr concentration was in accord with previously reported Cr incubation experiments [21].

For Cr and GAA synthesis experiments, astrocytes were incubated for 24 hours with [^15 ^N_2_]Arg and [^13 ^C_2_,^15^N]Gly at three different concentrations (5 mmol/L, 10 mmol/L, and 15 mmol/L). By contrast neuronal cerebellar granule cells were incubated with [^15 ^N_2_]Arg and [^13 ^C_2_,^15^N]Gly at the single concentration of 10 mmol/L. This was done in order to optimize the number of cells available for each experiment, given the fact that neurons proliferated much less than astrocytes. Labelled precursor concentrations were in accord with incubation experiments previously reported in lymphoblasts [12]. For each experiment a sample of cells incubated in medium without labelled precursors (control sample) was included.

After 24 hours of incubation the medium was aspirated, cells were washed twice with 10 ml of saline solution and detached from the flask's wall with trypsin. Afterwards, the cells were rapidly washed twice with 10 ml of saline solution by suspension and centrifugation at 1200 g for 10 min, to remove any contamination of labelled amino acids; pellets were then suspended into 100 μl of saline solution and stored frozen at -80°C until analysis. To lysate the cells, the pellets were frozen and thawed for six times at -70°C, placed in the ultrasonic bath for 30'and then centrifuged at 15000 g for 30'. The supernatant was recovered, an aliquot was used for protein assay (Bio-rad, Hercules, CA, USA) and the remaining was deproteinized using the Centricon filters at 5000 g for 50'. The cell extract was then analysed by ESI-MS/MS. The incubation experiments were replicated 3 times and the cell extracts were analysed twice.

### MS/MS analysis

ESI-MS/MS was used for the analysis of the cell extracts. In GAA and Cr synthesis experiments Gly, [^13 ^C_2_,^15^N]Gly, Arg, [^15 ^N_2_]Arg, GAA, [1,2-^13 ^C_2_,^15 ^N_3_]GAA, Cr and [1,2-^13 ^C_2_,^15^N_3_]Cr in the cell extract were simultaneously determined. For the study of CT1 expression Cr, D3Cr and GAA in the cell extracts were determined.

The cell extracts were diluted 1:5 with methanol/water (80/20 v/v) containing the internal standard [1,2-^13 ^C_2_]GAA in order to obtain a final concentration of 0.5 μmol/L. Then the sample was dried under a nitrogen flow at 45°C using an EvapArray Sample Concentator (Porvair Advanced Materials, UK) and derivatized to butyl esters using 3 mol/L HCl in n-butanol solution at 60°C for 30 min. After derivatization the sample was dried under a nitrogen flow at 45°C and then recovered with 100 μl of methanol/water solution (80:20) containing 0.1% formic acid. Twenty microliters of the obtained sample was injected for the ESI-MS/MS analysis.

An Agilent 1100 micropump (Agilent Technologies, Wilmington, DE, USA) and a Series 200 autosampler (Perkin Elmer, USA) were used for solvent delivery and automated sample introduction. Mobile phase was methanol/water (80:20) at a flow rate of 40 μl/min.

A triple quadruple mass spectrometer API 2000 (ABI Sciex, Toronto, Canada) equipped with a TurboIonSpray source was used. The data were acquired and processed using Analyst 1.1. Resolution and calibration adjustments of quadrupoles were made by direct infusion at a 10 μl/min of a 0.1 mmol/L polypropyleneglycole solution by an infusion pump. Q1 and Q3 were used at unit and high resolution respectively. Turbo ion spray source was operated in the positive ion mode at 350°C with a drying-gas at 20 psi and a capillary voltage of 5500 V. Nitrogen was used as curtain and collision gas. Multiple reaction monitoring was used for the detection of [M + H]^+ ^and product ions. The declustering potentials were 23, 27, 19 and 20 V, collision exit potential were 2.8, 2.0, 0.4, and 10 V and collision energy were 19, 26, 45 and 12 eV for GAA, Cr, Arg, Gly respectively and the same parameters were used for their isotopomers.

As result of the GAA and Cr synthesis experimental conditions, labelled and unlabelled precursors coexisted inside the cells and therefore we obtained enzyme products with a different number of stable isotopes (monolabelled, trilabelled and pentalabelled products). Since the higher concentration of the labelled in respect to unlabelled precursors, the pentalabelled forms ([1,2-^13 ^C_2_,^15^N_3_]GAA and [1,2-^13 ^C_2_,^15^N_3_]Cr) were prevalent. Therefore we selected them as reference compounds, also considering that their signals were far from isotopic distribution of the natural compounds.

**Figure 1:**
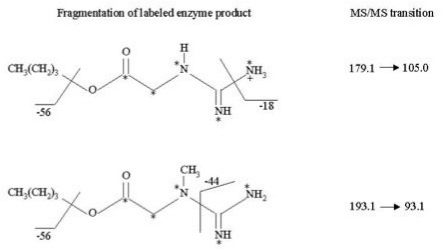
**Analysis by MS/MS: fragmentation by collision-induced dissociation (CID)**. Fragmentation pattern and selected ion transitions (m/z) of butylated [1,2-^13 ^C_2_,^15^N_3_]GAA and [1,2-^13 ^C_2_,^15^N_3_]Cr used for MS/MS analyses are shown.

The ion transitions for the labelled products [1,2-^13 ^C_2_,^15^N_3_]GAA and [1,2-^13 ^C_2_,^15^N_3_]Cr were defined upon the study of fragmentation of corresponding unlabelled compounds. The fragmentation of the protonated molecular ion of GAA, producing the fragment ion at 101.1 m/z, involves the loss of a molecule of butene and ammonia, while the formation of 90.1 m/z by protonated Cr, involves the loss of a molecule of butene and cyanamide (Figure 1). According to this considerations we used 105.0 and 93.1 m/z product ions for the quantification of respectively [1,2-^13 ^C_2_,^15^N_3_]GAA and [1,2-^13 ^C_2_,^15^N_3_]Cr.

The ion transitions were reported in Table 1. The quantitative determination of Gly, [^13 ^C_2_,^15 ^N]Gly, Arg, [^15 ^N_2_]Arg, Cr, [1,2-^13 ^C_2_,^15 ^N_3_]Cr and D3Cr were obtained using internal standard calibrations. For the construction of calibration curves four different concentrations of [^13 ^C_2_,^15^N]Gly, [^15 ^N_2_]Arg and D3Cr were used and the linear correlation coefficient was in all cases higher than 0.999. The isotope dilution method for the quantitation of labelled and unlabelled GAA was used.

**Table 1:** Parent and product ion transitions used for analysis by mass spectrometry

	Parent ion m/z	Product ion m/z	LOD (nmol/L)	LOQ (nmol/L)
Gly	132.1	76.1	-	-
[^13^C_2_,^15^N]Gly	135.1	78.6	8	30
Arg	231.2	70.2	-	-
[^15^N_2_]Arg	233.2	70.2	24	26
GAA	174.1	101.1	-	-
[1,2-^13^C_2_,^15^N_3_]GAA,	179.1	105.0	30	50
Cr	188.1	90.1	-	-
[1,2-^13^C_2_,^15^N_3_]Cr	193.1	93.1	5	8
D3Cr	191.2	93.1	2	3
[1,2-^13^C_2_]GAA (IS)	176.1	103.1	-	-

Parent and product ion transitions used in MRM and lower detection and quantitation limits are reported. The LOD and LOQ were estimated analyzing ten blank samples and were calculated adding to the mean concentration respectively 3 and 10 SD.

Linearity, recovery, within-run precision (data not shown), and sensitivity (the LOD and LOQ, Table 1) were studied demonstrating that this method was sensitive, accurate and precise.

### Real time PCR

RNA from different Rattus norvegicus (Sprague Dawley) tissues was obtained. RNA was isolated from Kidney, Liver, and astrocytes using RNeasy Mini Kit (QIAGEN, Milan, Italy), from cerebellar granule cells, using RNeasy Lipid Tissue Mini kit (QIAGEN, Milan, Italy) and from blood using QIAamp RNA Blood Minikit (QIAGEN, Milan, Italy) as described from manufacturer. cDNA synthesis from total RNA was performed with High Capacity cDNA Archive Kit (Applera Italia, Monza, Italy). The resulting cDNA template, at a concentration of 50 ng/μl, was subjected to relative-quantitative Real-time PCR using Taqman gene expression assays (Applera Italia, Monza, Italy), the TaqMan Universal PCR Master Mix (Applera Italia, Monza, Italy) and the Two-Step Real-Time PCR System (Applera Italia, Monza, Italy). Quantitative Real Time PCR analysis of AGAT, GAMT and SLC6A8 mRNA expression were carried out in a Two-Step Real-Time PCR System using the TaqMan Rat Gene Expression Assays Rn00562952_m1 for target gene GAMT (EC 2.1.1.2), Rn00578954_m1 for target gene AGAT (EC 2.1.4.1), Rn00506029_m1 for target gene SLC6A8 (Solute carrier family 6 member 8) and Rn99999916_s1 for the internal normalization gene GAPDH (Applera Italia, Monza Italy). For quantitative Real Time PCR analysis, each sample was run in triplicates and the entire analysis was confirmed at list twice. Each run included a no template control (NTC) to test for contamination of assay reagents. After an initial Amperase UNG Activation at 50°C for 2 min, and a 95°C denaturation for 10 min, the reactions were cycled 40 times with a 95°C denaturation for 15 s, and a 60°C annealing for 1 min. Three types of controls aimed at detecting genomic DNA contamination in the RNA sample or during the Reverse Transcription (RT) or quantitative Real Time PCR reactions were always included: a RT mixture without reverse transcriptase, a RT mixture including the enzyme but no RNA, negative control (reaction mixture without cDNA template). Relative quantification was performed using the comparative threshold (CT) method after determining the CT values for reference (GAPDH) and target (AGAT, GAMT and SLC6A8) genes in each sample sets according to the 2^-ΔΔCT ^method. Changes in mRNA expression level were calculated after normalization to GAPDH. As calibrator sample we used cDNA from arbitrarily selected control cell line or tissue.

### Statistical analysis

Unless otherwise indicated, all data are presented as the mean ± SD. Student's *t*-test was used to determine the significance of differences between two group means. The relationship between two variables was tested using linear regression analysis. For statistical evaluation Statistica (StatSoft, Tulsa, USA) was used.

## Results

### Cr uptake

**Figure 2:**
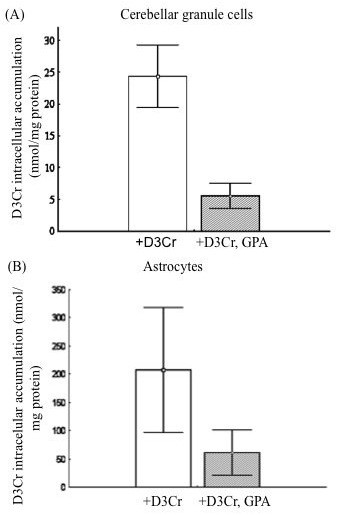
**Functional expression of CT1 in rat cerebellar granule cells and astrocytes**. Incubation of rat cerebellar granule cells (A) and astrocytes (B) with 1 mmol/L D3Cr in culture medium in presence and absence of 1 mmol/L GPA: intracellular D3Cr mean values are given along with standard deviation for three different experiments (each sample was analysed two times). The addition in culture medium of GPA resulted in a marked inhibition of D3Cr uptake (77% and 68% respectively in cerebellar granule cells and astrocytes). D3Cr uptake in absence of GPA was significantly higher than that in presence of GPA, showing the occurrence of Cr active transport, in cerebellar granule cells as well as in astrocytes (paired Student's *t*-test, N = 6, p < 0.05). Difference in D3Cr uptake between the two cell types was not statistically significant (unpaired Student's *t*-test, N = 6, p = 0.17).

To investigate the functional expression of CT1 in cerebellar granule cells and astrocytes these cells were incubated with D3Cr, and its uptake was assessed in the presence or absence of GPA. After 24 hours of incubation with 1 mmol/L D3Cr, both cerebellar granule cells and astrocytes showed a high intracellular accumulation of the labelled compound (24.4 ± 5.3 and 207.5 ± 119.6 nmol/mg protein, respectively). These values were as an average 10 and 122 times higher, respectively, than those of the endogenous (unlabelled) Cr, that were 2.5 ± 0.9 and 1.7 ± 0.6 nmol/mg protein, respectively in rat cerebellar granule cells and astrocytes. The addition of GPA to the culture medium resulted in 77 ± 5% and 68 ± 1% inhibition of the D3Cr uptake in cerebellar granule cells and astrocytes, respectively (Figure 2) and the difference in D3Cr accumulation in the presence and absence of GPA was significantly different (paired *t*-test, N = 6, p = 0.006 and p = 0.039 for granule cells and astrocytes, respectively), thus demonstrating that the uptake was largely dependent on the integrity of the Cr transporter.

### Cr synthesis in rat cerebellar granule cells

Intracellular precursor accumulation, as well as GAA and Cr synthesis were investigated by comparing granule cells incubated with and without [^13 ^C_2_,^15 ^N]Gly and ^15 ^N_2_-Arg, (10 mM). Results are summarized in Table 2. After 24 hours of incubation: a) the concentration of ^15 ^N_2_-Arg was 56.3 ± 20.4 nmol/mg protein and was similar to that of the endogenous, unlabelled Arg (55.3 ± 6.5 nmol/mg protein); b) [^13 ^C_2_,^15 ^N]Gly concentration was 710.5 ± 216.1 nmol/mg protein and was higher than unlabelled Gly (137.9 ± 29.8 nmol/mg protein); c) [^13^C_2_^15^N_3_]GAA concentration was 7.4 ± 3.2 nmol/mg protein, which was 34 times the concentration of unlabelled GAA (0.22 ± 0.05 nmol/mg protein). [^13^C_2_^15^N_3_]GAA signal was 62 times the corresponding signal due to the background noise in control cells; d) [^13^C_2_^15^N_3_]Cr concentration was 1.6 ± 0.4 nmol/mg protein, 2.6% of the concentration of unlabelled endogenous Cr (62.2 ± 15.3 nmol/mg protein). [^13^C_2_^15^N_3_]Cr signal was 40 times the corresponding signal due to the background noise in control samples.

**Table 2:** GAA and Cr synthesis experiments in rat cerebellar granule cells

Concentration of [^15^N_2_]Arg and [^13^C_2_,^15^N]Gly in the medium (mmo1/L)	[^15^N_2_]Arg in the cells (nmol/mg protein)	Arg in the cells (nmol/mg protein)	[^15^N_2_]Arg/Arg
0	1.6 ± 0.8	153.4 ± 105.3	0.009 ± 0.001
10	56.3 ± 20.4 ^NS^	55.3 ± 6.5	1.00 ± 0.25

	[^13^C_2_,^15^N]Gly in the cells (nmol/mg protein)	Gly in the cells (nmol/mg protein)	^13^C_2_,^15^N]Gly/Gly

0	1.8 ± 1.2	470.8 ± 232.7	0.007 ± 0.004
10	710.5 ± 216.1 **	137.9 ± 29.8	5.08 ± 0.68

	[^13^C_2_^15^N_3_]GAA in the cells(nmol/mg protein)	GAA in the cells (nmol/mg protein)	[^13^C_2_^15^N_3_]GAA/GAA

0	0.12 ± 0.07	2.08 ± 1.43	0.085 ± 0.029
10	7.4 ± 3.2 *	0.22 ± 0.05	33.3 ± 7.9

	[^13^C_2_^15^N_3_]Cr in the cells (nmol/mg protein)	Cr in the cells (nmol/mg protein)	[^13^C_2_^15^N_3_]Cr/Cr

0	0.04 ± 0.02	261.3 ± 138.4	< 0.001
10	1.6 ± 0.4**	62.2 ± 15.3	0.027 ± 0.007

Intracellular concentrations of Gly, [^13^C_2_,^15^N]Gly, Arg, [^15^N_2_]Arg, GAA, [1,2-^13^C_2_,^15^N_3_]GAA, Cr, and [1,2-^13^C_2_,^15^N_3_]Cr after 24 hours in vitro incubation with 10 mmol/L [^15^N_2_]Arg and 10 mmol/L [^13^C_2_,^15^N]Gly. Zero concentration in the first column (first line) corresponds to cells incubated in medium without labeled precursors. The value reported of labeled compounds is due to background noise. The statistical significance of the differences between intracellular concentrations of labeled and unlabeled compounds was assessed by paired Student's *t*-test: NS not significant, * p < 0.05, ** p < 0.01. The intracellular concentration of [^13^C_2_,^15^N]Gly was significantly higher than that of Gly and the concentration of [^15^N_2_]Arg was similar to that of Arg. Accordingly, the intracellular [^13^C_2_^15^N_3_]GAA concentration was significantly higher than GAA concentration whereas [^13^C_2_^15^N_3_]Cr is significantly lower than Cr. The ratios express the relative intracellular availability of labeled and unlabeled forms (right column).

### Cr synthesis in rat astrocytes

Astrocytes were incubated for 24 hours with 3 different concentrations of [^15 ^N_2_]Arg and [^13 ^C_2_,^15^N]Gly (5, 10 and 15 mmol/L). Results are summarized in Table 3.

**Table 3:** GAA and Cr synthesis experiments in rat astrocytes

Concentration of [^15^N_2_]Arg and [^13^C_2_,^15^N]Gly in the medium (mmol/L)	[^15^N_2_]Arg in the cells (nmol/mg protein)	Arg in the cells (nmol/mg protein)	[^15^N_2_]Arg/Arg
0	1.8 ± 1.1	172.0 ± 78.2	0.01 ± 0.00
5	137.9 ± 71.6 ^NS^	145.5 ± 73.4	1.12 ± 0.52
10	504.0 ± 390.0 *	178.6 ± 137.9	3.43 ± 1.66
15	957.4 ± 500.45 **	215.5 ± 132.5	6.74 ± 4.72

	[^13^C_2_,^15^N]Gly in the cells (nmol/mg protein)	Gly in the cells (nmol/mg protein)	[^13^C_2_,^15^N]Gly/Gly

0	2.8 ± 0.9	365.7 ± 43.2	0.01 ± 0.00
5	1003.8 ± 1083.3 ^NS^	696.3 ± 604.2	1.28 ± 0.49
10	2205.5 ± 2382.0 *	893.8 ± 958.4	2.53 ± 0.22
15	3213.9 ± 2482.9 *	744.8 ± 512.7	4.43 ± 0.85

	[^13^C_2_^15^N_3_]GAA in the cells (nmol/mg protein)	GAA in the cells (nmol/mg protein)	[^13^C_2_^15^N_3_]GAA/GAA

0	0.25 ± 0.09	1.46 ± 1.46	0.29 ± 0.14
5	10.34 ± 7.90 *	2.37 ± 2.23	4.47 ± 3.97
10	17.18 ± 13.36 *	1.88 ± 1.87	13.30 ± 14.32
15	21.59 ± 15.00 **	1.64 ± 1.11	18.18 ± 17.96

	[^13^C_2_^15^N_3_]Cr in the cells (nmol/mg protein)	Cr in the cells (nmol/mg protein)	[^13^C_2_^15^N_3_]Cr/Cr

0	0.10 ± 0.05	100.4 ± 118.7	0.00 ± 0.00
5	12.13 ± 12.64 *	86.53 ± 71.65	0.12 ± 0.04
10	10.88 ± 8.48 **	59.48 ± 23.72	0.17 ± 0.08
15	11.00 ± 6.85 **	48.54 ± 10.58	0.21 ± 0.09

Intracellular concentrations of Gly, [^13^C_2_,^15^N]Gly, Arg, [^15^N_2_]Arg, GAA, [1,2-^13^C_2_,^15^N_3_]GAA, Cr and [1,2-^13^C_2_,^15^N_3_]Cr after 24 h in vitro incubation with [^15^N_2_]Arg and [^13^C_2_,^15^N]Gly under different concentrations of labeled precursors in the medium: no labeled precursors (0), 5, 10 and 15 mmol/L. The statistical significance of the differences between intracellular concentrations of labeled and unlabeled compounds was assessed by paired Student's *t*-test: NS not significant, * p < 0.05, ** p < 0.01. The concentrations of [^13^C2,^15^N]Gly and [^15^N_2_]Arg were significantly higher than those of Gly and Arg (except for cells incubated with [^15^N_2_]Arg mmol/L), showing that the labeled precursors are highly available into the cells for Cr synthesis. [^13^C_2,_^15^N_3_]GAA concentrations were also significantly higher than those of GAA, whereas [^13^C_2_,^15^N_3_]Cr levels remained significantly lower than those of Cr. The ratios express the relative intracellular availability of labeled and unlabeled forms (right column).

The intracellular levels of the labelled amino acids paralleled those in the medium and, except for the concentration of 5 mmol/L, were higher than the concentrations of unlabelled Arg and Gly, thus demonstrating their uptake by astrocytes.

**Figure 3:**
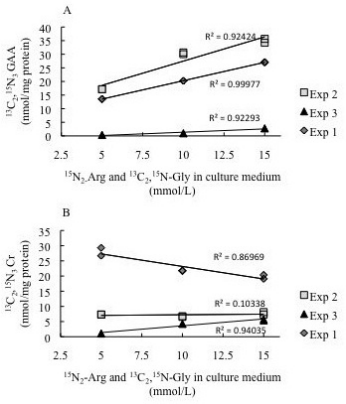
**Functional expression of GAMT and AGAT in rat astrocytes**. Intracellular [^13 ^C_2_,^15^N_3_]GAA (A) and [^13^C_2_,^15^N_3_]Cr (B) concentrations in function of [^13^C_2_,^15^N]Gly and[^15 ^N_2_]Arg concentrations in culture medium, in three sets of cell samples (experiment 1, 2 and 3) are reported. To test the correlation among the intracellular concentration of labelled products and the precursor concentrations in culture medium, linear regression analysis was performed. Whereas there was a linear correlation for labelled GAA (p < 0.0022), no clear and consistent linear correlation for labelled Cr was found. Both graphs show that the cultures of experiment 3 yielded a lesser amount of GAA and Cr. Inspection of the cultures did not provide any obvious explanation for this result.

After incubation with labelled precursors, the [^13^C_2,_^15^N_3_]GAA signal in cell extract was strongly increased, reaching 86 times the corresponding background noise in control samples. [^13^C_2,_^15^N_3_]GAA concentration increased proportionally with the concentration of labelled precursors in the medium (R^2 ^> 0.9229; p < 0.002; Figure 3a). The ratio between [^13^C_2,_^15^N_3_]GAA and the unlabelled, endogenous, GAA reached 18 (mean value) for the highest precursor concentration.

We found also a relevant increase of [^13^C_2_^15^N_3_]Cr, whose signal was significantly higher (up to 121 times higher) than the corresponding signal in control samples. No correlation was found between the [^13^C_2_^15^N_3_]Cr levels and those of labelled precursors in the medium (Figure 3b) nor with intracellular [^13^C_2_^15^N_3_]GAA. The concentration of [^13^C_2_^15^N_3_]Cr did not exceed 21% (mean value) of that of unlabelled endogenous Cr.

### Gene expression

**Figure 4:**
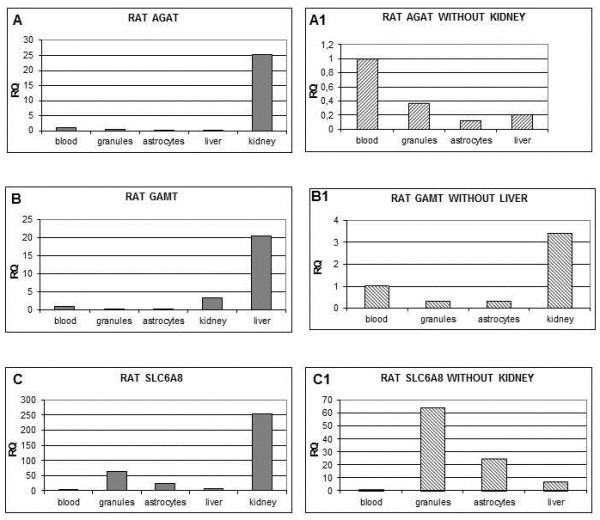
**Relative quantification of gene expression using Real Time PCR**. Blood expression level is used as calibrator (Relative quantification (RQ) = 1). Panels at right side (A1, B1, C1) are a magnification of those on the left side (A, B, C) excluding tissues with higher expression level.

To get information about the level of gene expression in rat astrocytes and cerebellar granule cells we used a PCR-Real Time relative quantification. To this aim, we compared AGAT, GAMT and SLC6A8 gene expression in different tissues such as blood, kidney and liver. Figure 3 shows that AGAT and SLC6A8 transcripts were highly expressed in the kidney and GAMT in the liver, while a very low SLC6A8 expression was found in blood cells. Leukocytes expression was used as "calibrator" for the relative assessment of each gene, i.e. the amount of gene expression in blood cells was used as unit for the quantification of the expression in the other tissues. Among the tissues analyzed, after excluding the kidney in which it was very high (Figure 4, right panel), AGAT gene expression was higher in blood cells than in the liver (about 4/1). AGAT was about 1/3 lower in astrocytes than in cerebellar granule cells (p < 0.005) which, in turn, expressed 37% of blood cells.

GAMT gene was equally expressed in cerebellar granule cells and astrocytes (31 and 33% of blood cell expression, respectively). We found that GAMT expression in the kidney was 3.5 times higher than in blood cells. SLC6A8 was about 3 times more expressed in cerebellar granule cells than in astrocytes (p < 0.005). In order to test the possible influence of experimental conditions on gene expression, SLC6A8 transcripts were evaluated in P1, P2 and P3 generations of astrocytes by real time PCR. A stable and overlapping expression was found along the three cell generations (relative quantification expressed as log_10 _values were 2.06 ± 1.61, 2.09 ± 1.66 and 2.09 ± 1.68 respectively for P1, P2 and P3 generations).

## Discussion

Cellular incorporation of stable isotope from nutrient into downstream metabolites is largely used for the assessment of metabolic fluxes and the step of quantifying labelled and unlabelled molecules can be achieved by mass spectrometry, with the advantages of excellent sensitivity and linear dynamic range, and simultaneous detection of precursors, products, and associated isotopomers [22]. Labelled Arg and Gly have been successfully used to assess AGAT activity in lymphocytes and lymphoblasts [23] by gas-chromatography-mass spectrometry. Using this approach we have developed a new ESI-MS/MS method and investigated Cr uptake and synthesis in rat cerebellar granule cells and astrocytes by incubating the cells with either labelled Cr or with labelled Arg and Gly, which are the precursors of GAA and Cr.

The converging results of our functional and gene expression studies showed that in the rat both neurons and astrocytes are capable to express the enzymatic machinery to both uptake and synthesize Cr.

### Cr uptake

Cr uptake in our study was greatly reduced by GPA, an inhibitor of CT1 [1,24], thus strongly suggesting that it mostly happens through this transporter. This functional result was supported by gene expression analysis, showing that both cell types express CT1. We confirmed previous data showing that adult rat cerebellar granule cells express the Cr transporter [15,25-27], that contributes in a relevant way to the whole intracellular pool of this compound. A low expression of Cr transporter in the rat brain soon after the birth and a rapid increase during the first weeks of life was reported by some studies [25], while others detected an earlier expression of Cr transporter in embryonic CNS [28]. By contrast, Moller and Hamprecht [29] found only a feeble Cr uptake in neuroblastoma cell line and no Cr transport in neuron-rich primary cultures from embryonic rat. Thus, our finding adds to the existing store of partially conflicting results.

Concerning Cr uptake by the astrocytes, our results confirm those of a previous study based on a kinetic approach [29]. On this topic, too, conflicting results are reported in the literature. CT1 was detected in perivascular astrocytes of the retina [30] and cerebellum [31], but not in other regions of CNS [31], while no CT1 expression was found in astrocytes by in situ hybridization [15,27]. Braissant et al. [21] showed that protracted exposure to very high levels of NH4Cl induced SLC6A8 transcription and translation in astrocytes, where it became then detectable by in situ hybridization and immunohistochemistry, thus suggesting that the SLC6A8 gene may be differentially expressed under different experimental conditions. To the best of our knowledge, NH4Cl is the only factor that has been proven to induce expression of the SLC6A8 gene [21]. However, the reliability of our results is supported by the stability of SLC6A8 transcription in three generations of cultured astrocytes (P1, P2 and P3), suggesting that SLC6A8 expression is not affected by the in vitro condition.

As a word of caution, while our findings show that astrocytes have the capability to express the Cr transporter, and to uptake Cr, we can not rule out that this capability may be differently expressed in different conditions.

### Cr synthesis

Our findings show that both granule cells and astrocytes are capable to uptake Arg and Gly and to use them to synthesize GAA and Cr. Again, these functional findings are supported by gene expression analysis, showing that both cell types express AGAT and GAMT, the enzymes that are required to synthesize Cr from Arg and Gly, which have their own transporters at the blood brain barrier and on the plasma membrane of nervous cells [32-35]. We found that labelled Gly was taken up by both astrocytes and neurons in a quantity larger than labelled Arg (Table 2 and 3). That probably depends on different characteristics of the corresponding transporters, whose investigation however was outside the scope of our paper. Previous studies demonstrated GAMT activity in neuroblastoma cell lines [16]. They also demonstrated Cr synthesis from Gly in astroglial cells (at a rate dependent on Arg and Methionine levels in the substrate [17]), and in brain cell 3D cultures (that are able to convert the GAA taken-up from medium into Cr [36]). Moreover, AGAT and (to a lesser extent) GAMT mRNA were ubiquitously found in the adult rat brain by in situ hybridization [15].

### Cr synthesis vs. Uptake

An important difference arises in our data between Cr synthesis and uptake when we consider the magnitude of the results. Cr uptake was in our hands a very efficient process in both granule cells and astrocytes. After incubation with labelled Cr the 91% and 99% of the total intracellular Cr content, respectively in neurons and in astrocytes, is due to transport. This effect is very robust and sizable. By contrast, after 24 hours cell incubation with high concentrations of Cr precursors Arg and Gly, the *de novo *synthesized Cr was only about 2.7% (in the case of neurons) and 12-21% (in the case of astrocytes). The remaining unlabelled Cr could be ascribed to pre-existing intracellular Cr pool, to calf serum in culture medium (see methods) and to *de novo *synthesis starting from unlabelled precursors. However the contribution of unlabelled precursors to Cr synthesis is relatively negligible since their intracellular concentrations were minor or equal to labelled ones. Furthermore, it should be noted that the intermediate labelled product GAA was highly available for Cr synthesis in both cell types, increasing 33 times in neurons and up to about 18 times in astrocytes (Tables 2 and 3). In astrocytes it was possible to study the relationship between precursors concentration and GAA or Cr content. A linear relationship was found for GAA, while no such relationship was found for Cr. Taken together, these results suggest that in brain cells both Cr synthesis and uptake are possible, and that under the in vitro conditions we used the latter is more efficient than the former. Moreover, they suggest that the most critical step in the synthesis of Cr by brain cells is the conversion of GAA into Cr, both in neurons and in astrocytes. We do not exactly know why the conversion of GAA into Cr is so limited. Tachikawa et al. [18] demonstrated by an immunohistochemical approach a preferential glial expression of GAMT coupled with a preferential neuronal expression of ubiquitous mitochondrial CK in the adult mouse brain. This pattern of enzyme segregation suggested that neuronal Cr is supplied to nervous cells by local glial cells [18], a hypothesis later supported by some of us [37]. In this complex homeostatic model, which mimics the general separation between sites of Cr synthesis and utilization outside the CNS [1], the possible pivotal role of GAA remains to be clarified. Apart from endogenous synthesis, GAA is up taken by nervous cells via Cr [18] and Taurine [38] transporters and in brain parenchyma it is converted to Cr even if in a low proportion (29%) [36].

This issue was further addressed by exploring the co-expression of AGAT, GAMT and SLC6A8 proteins in rat grey matter cells [27,36]. These studies suggested that GAA and Cr synthesis may occur in different cells, and that GAA needs to be transferred between cells to be converted into Cr. By providing functional evidence that the conversion of GAA into Cr by brain cells is a quantitatively limited process, our findings do provide some support to that hypothesis. Alternatively, we should not underestimate the possible limiting effect of the availability of other metabolites involved in Cr homeostasis, such as S-adenosylmetionine [29], even if the limiting effect of this metabolite on Cr synthesis is considered not relevant under normal condition [1].

### Clinical implications

The presence of CT1 in both astrocytes and cerebellar granule cells, as well as in the endothelium of brain capillaries [39], and in choroid plexus during the embryonic development [28] strongly supports the view that blood Cr supply is an important source of brain Cr in physiological conditions. This conclusion is compatible with the metabolic pattern detected in patients affected by CT1 deficiency [9,40] as well as with the restoration of brain Cr under Cr supplementation observed in the defects of Cr synthesis: in AGAT deficiency (differently from GAMT deficiency, where GAA accumulates and competes with Cr for the CT1 transporter) 40% and 80% restoration of normal brain Cr level was obtained after 3 and 9 months of treatment, respectively [9,11]. A dosage as low as 100 mg/kg/bw/day was sufficient to replenish 60% of brain Cr and prevents the emergence of the disease in an early diagnosed and treated child with AGAT deficiency [9]. This support the view that Cr transport is probably much more efficient during fetal to perinatal and postnatal stages [28]. The increase of brain Cr observed in normal subjects under protracted Cr loading is much less relevant (3.5-13.3%), notwithstanding the concomitant increase of blood Cr from 50 to 800 μmol/L [41]. Similar results, which have been confirmed in animal models [42-44], overall support the view that Cr pool in CNS is subjected to a homeostatic control.

The above results may offer an explanation of the conflicting results that were obtained in patients affected by CT1 deficiency when treated with the Cr precursors in order to stimulate the endogenous synthesis of Cr. Our data suggest that Cr synthesis by brain cells may be limited, in such a way to possibly provide only a limited amount of the Cr that is needed by the brain.

Some limitations inherent to our study suggest caution in extrapolating our results to the in vivo conditions. In fact, we investigated only two kinds of cells from the cerebellum, while different brain regions and cells could be differently equipped in term of CK activity and related Cr availability [31]. Moreover, the need of large supplementation of Cr in patients with deficient Cr synthesis, and normal or increased CSF Cr - but not brain ^1 ^H-MRS Cr - in few CT1 deficient patients before [45] or during Cr treatment [5,13], remain to be explained. The recently developed Cr transporter knockout mice, that mimics several aspects of the human disease, will provide an exciting opportunity to verify different models of Cr transport and synthesis inside the CNS [46].

Summing up, while further research is needed to fully understand to what extent these in vitro results are relevant to the in vivo situation, our results show that both astrocytes and neurons possess the capability to both synthesize and uptake Cr, but that the latter is probably more robust than the former.

## Abbreviations

Arg: arginine; AGAT L-arginine:glycine amidinotransferase; Cr: creatine; [^13 ^C_2_,^15 ^N]Gly: [^13 ^C_2_,^15 ^N]glycine; CT1: creatine transporter; D3Cr: D_3_-creatine; GAA: guanidinoacetic acid; GAMT: S-adenosylmethionine:guanidinoacetate N-methyltransferase; Gly: glycine; GPA: β-guanidinopropionate; [^15 ^N_2_]Arg: L-[guanido-^15 ^N_2_]arginine; SLC6A8: creatine transporter gene; CNS: central nervous system; ESI-MS/MS: electrospray ionization tandem mass spectrometry; [1,2-^13 ^C_2_]GAA: ^13^C_2_-guanidinoacetic acid; [^13 ^C_2_,^15^N_3_]GAA: [1,2-^13 ^C_2_,^15^N_3_]guanidinoacetic acid; [^13 ^C_2_,^15^N_3_]Cr: [1,2-^13 ^C_2_,^15^N_3_]creatine.

## Competing interests

The authors declare that they have no competing interests.

## Authors' contributions

All Authors have each made a substantial contribution so as to qualify for authorship.

Specifically: a) CC, SS, and IA set up the new MS/MS methods for the intracellular assessment of labelled and unlabelled metabolites. They also were involved in the development of the experimental design and cooperated in the interpretation of the metabolic results; b) CC and CA performed genetic and metabolic molecular studies, interpreted genetic results and cooperated in the discussion concerning the linkage between biochemical and molecular results; c) EA, ST, EG, MR, and TF developed the in vitro model; arranged and accomplished cell coltures with labelled Cr and Cr precursors; d) VL and MB conceived and developed the experimental design, addressed the discussion on the interpretation of the results and coordinated the collaborative study. All authors read and approved the final manuscript.
